# MLPA analysis of transcriptional gene(s) variations in patients with congenital heart septation defects

**DOI:** 10.1186/1755-8166-7-S1-P23

**Published:** 2014-01-21

**Authors:** Rajasekhar Moka, Neetha John, Yashvanthi Borkar, K Ranjan Shetty

**Affiliations:** 1 School of Life Sciences, Manipal University, Manipal-576104, India

## Background

Congenital heart diseases (CHD) occur in ~1% live births and are among the most common birth defects. It accounts nearly about 46% of all deaths from congenital malformations. Adults with repaired congenital heart disease represent a complex and heterogeneous group of patients that are increasingly surviving beyond childhood. Advances in our molecular understanding of normal heart development have led to the identification of numerous genes necessary for cardiac morphogenesis. The etiological factors of many genetic syndromes and familial CHD have been identified, but the genetic basis of majority of them remains unknown. Objective of this study was to screen the transcriptional genes for novel mutations since majority of these involved in the early stages of cardiogenesis.

## Materials and method

Multiplex Ligation-dependent Probe Amplification (MLPA) was employed in to detect copy number changes in 50 patients’ DNA using the P311-A1 CHD SALSA MLPA Kit for the genes NKX2-5, GATA4, TBX5, BMP4 and CRELD1 in 25 genomic regions.

## Results

Copy number changes were identified in four (8%) two heterozygous deletions in BMP4 and TBX5 were observed in common in two patients atrial septum defects.

## Conclusions

MLPA assay can be used for detection of copy number changes in patients with non-syndromic CHDs as a first line of screening test.

